# On gardens, brackets and money

**DOI:** 10.1590/2177-6709.21.2.010-011.edt

**Published:** 2016

**Authors:** Leandro Silva Marques

**Affiliations:** 1 Adjunct professor of Orthodontics, Universidade Federal dos Vales do Jequitinhonha e Mucuri (UFVJM), Diamantina, Minas Gerais, Brazil. Provost of Administration, Universidade Federal dos Vales do Jequitinhonha e Mucuri (UFVJM), Diamantina, Minas Gerais, Brazil.

*"As long as the roots are not severed, all is well.*

*And all will be well in the garden. (...) Growth has its seasons."*

*Chance, a gardener from the movie Being There, played by Peter Sellers.*

Understanding the scenarios of the orthodontic community in different countries may favor the political and administrative positioning of institutions involved with Orthodontics as a science.

In the movie *Being There* (1979), Peter Sellers plays the gardener Chance. Living completely secluded in Washington D.C., what Chance knows about the world is what he sees on television. He entered into the real world due to misfortune and ends up joining an exclusive circle of powerful government men who strive for his "wisdom." Brazil was once a huge garden. Orthodontists lived as gardeners "In the land of the blind, the one-eyed man is king" style. Suddenly, dark times came along, plagues and storms decimated all the splendor of the garden and darkness fell. Perhaps you might find this story a somber one, but I urge you to read on. Paraphrasing Nietzsche: "There are no facts, there are only interpretations."

Being an orthodontist in Brazil in the 1970s, 80s and early 90s was synonymous with social prestige, offices packed with patients and plenty of financial gains. There were few orthodontists, few specialization courses and an enormous demand for orthodontic treatment. Access to the benefits provided by the use of an orthodontic appliance was mediated solely by the ability to afford treatment and was considered a status symbol. In this context, the number of dental schools boosted from 90 to 202 in the last 10 years for a population of 200 million inhabitants. To put this into perspective, there are 280 million people living in the USA, and there was a decline from 65 to 58 in the number of institutions within the same period. Meanwhile, in Brazil, there was an indiscriminate proliferation of new specialization courses in Orthodontics associated with a veritable plethora of nonexpert dentists installing brackets without any type of criteria. The outcomes of all this was the popularization of orthodontic treatment in Brazil allied with the impossibility on the part of the general public in choosing a true specialist. Thus, treatment became more affordable, but with dubious quality; and a specialty once seen as glamorous began to be eyed with distrust. 

On the other hand, the adverse effects of the crisis in Brazilian Orthodontics have contributed above all to significant changes. Organizations, such as the *Brazilian Association of Orthodontics* (ABOR), are mobilizing support to establish criteria for the training of new specialists and the recognition of courses that are already in operation. There was the establishment of the *Brazilian Board of Orthodontics* and public awareness campaigns have been carried out regarding the importance of choosing a duly qualified oral healthcare professional to perform orthodontic treatment. Furthermore, there has been an increasing amount of scientific production in the field of Orthodontics in Brazil. To give the reader an idea, among the 1465 publications in AJO-DO in the last five years, 98 (7%) were from Brazilian authors. When considering the last three years, *Dental Press Journal of Orthodontics* is the Brazilian dental journal with the highest number of publications.

In Brazil, as in the rest of the world, the seasons have changed. Orthodontists are faced with the challenge of recycling their values and paradigms. In times of clinical decision making based on evidence, it is necessary to rethink the ethical and moral values that guide our profession. Perhaps the profits are no longer what they used to be in the past, but reflection is needed on the fact that changes do not necessarily occur for the worse, as Chance, the gardener, implied in the epigraph thereof.

Leandro Marques - Adjunct editor 

(lsmarques.prof@gmail.com)

## Special issue on SYSTEMATIC REVIEWS

Researchers, graduate students and specialists are invited to submit their manuscripts to the special themed issue on Systematic Reviews (SisRev). Articles fitting into this category (SisRev) will be given preference to join the issue - after being peer-reviewed, following previously established standards.

Manuscript submission deadline for the special issue ends on the 31^st^ of May, 2016.

All selected articles will be published on the Nov./Dec. 2016, vol. 21, n. 6 issue.

We are waiting for you. 

David Normando - editor-in-chief (davidnormando@hotmail.com)

